# Cinchonia Alkaloid

**Published:** 1878-10-20

**Authors:** 


### CINCHONIA ALKALOID.

Is it true that Cinchonia can be relied upon as a substitute for the sulphate of Quinia? The increasing scarcity and high price of the latter article gives importance to this inquiry, especially at the present time. We have before had occasion to refer to this subject, and gave the result of experiments with this article, highly favorable to its efficacy as a tonic and antiperiodic.

In our March number, page 90, a medical gentleman to whom we handed a sample, sent us by Messrs Powers & Wightman, tested the remedy with satisfsctory results in two cases of malarial fever, and also relieved with it a case of obstinate menorrhagia, which it cured by its tonic property, and its power of stimulating the capillaries and equilizing the circulation. The preparation used was that lately introduced by Messrs Powers & Wightman, of cinchonia combined with sugar of milk and the bicarb of soda. It was found that thus combined the cinchonia is not only antiperiodic but anodyne in n its effects upon the nervous system.

The same Practitioner informs me that he has subsequently used the cinchonia uncombined in intermittent fevers with uniform success.

We have been using the cinchonia of late in lieu of the sulphate of quinia and find it equally efficient as a tonic and antiperiodic. It is almost tasteless and may be readily administered to children. We have not observed any unpleasant effect upon the ears or tendency to nausea. Dr. T. S. Powell—senior editor of this journal—has used the cinchonia with good success in intermittents, and has found it to be useful in solution with an excess of the aromatic sulphuric acid in menorrhagia, particularly in analmic and relaxed habits. A favorite formula with him in menorrhagia is the following:

| | |
|---|---|
| R. Cinchonia | grs. xxv |
| Simple Elixir | oziv |
| Aromatic Sulphuric acid | driij |
| F ext. ergot | driij |

Dose, one teaspoonful three to four times a day. In the absence of the simple elixir water may be used and a little sugar or syrup added.

A medical gentleman of Dekalb county, Ga., informs us that he has used the cinchonia for several years in doses a little larger than those of the sulphate of quinine and has found it equally efficient in the treatment of fevers. We have similar information from another practitioner who has sent us a communication on the subject which we expect to publish in our next.

The drug is certainly much more pleasant to the taste than the sulphate of quinine and being much cheaper it would seem that it ought to come into general use.

